# Radiation-induced double-strand breaks by internal *ex vivo* irradiation of lymphocytes: Validation of a Monte Carlo simulation model using GATE and Geant4-DNA

**DOI:** 10.1016/j.zemedi.2023.07.007

**Published:** 2023-08-18

**Authors:** Maikol Salas-Ramirez, Lydia Maigne, Giovanna Fois, Harry Scherthan, Michael Lassmann, Uta Eberlein

**Affiliations:** a Department of Nuclear Medicine, University of Würzburg, Würzburg, Germany; b Laboratoire de Physique de Clermont, University of Clermont Auvergne, Clermont, France; c Bundeswehr Institute of Radiobiology affiliated to the University of Ulm, Munich, Germany

**Keywords:** Monte Carlo simulation, Geant4-DNA, GATE, Blood dosimetry, DNA damage

## Abstract

This study describes a method to validate a radiation transport model that quantifies the number of DNA double-strand breaks (DSB) produced in the lymphocyte nucleus by internal *ex vivo* irradiation of whole blood with the radionuclides ^90^Y, ^99m^Tc, ^123^I, ^131^I, ^177^Lu, ^223^Ra, and ^225^Ac in a test vial using the GATE/Geant4 code at the macroscopic level and the Geant4-DNA code at the microscopic level.

**Methods:**

The simulation at the macroscopic level reproduces an 8 mL cylindrical water-equivalent medium contained in a vial that mimics the geometry for internal *ex vivo* blood irradiation. The lymphocytes were simulated as spheres of 3.75 µm radius randomly distributed, with a concentration of 125 spheres/mL. A phase-space actor was attached to each sphere to register all the entering particles. The simulation at the microscopic level for each radionuclide was performed using the Geant4-DNA tool kit, which includes the *clustering* example centered on a density-based spatial clustering of applications with noise (DBSCAN) algorithm. The irradiation source was constructed by generating a single phase space from the sum of all phase spaces. The lymphocyte nucleus was defined as a water sphere of a 3.1 µm radius. The absorbed dose coefficients for lymphocyte nuclei (d_Lymph_) were calculated and compared with macroscopic whole blood absorbed dose coefficients (d_Blood_). The DBSCAN algorithm was used to calculate the number of DSBs. Lastly, the number of DSB∙cell^−1^∙mGy^−1^ (simulation) was compared with the number of radiation-induced foci per cell and absorbed dose (RIF∙cell^−1^∙mGy^−1^) provided by experimental data for gamma and beta emitting radionuclides. For alpha emitters, d_Lymph_ and the number of α-tracks∙100 cell^−1^∙mGy^−1^ and DSBs∙µm^−1^ were calculated using experiment-based thresholds for the α-track lengths and DSBs/track values. The results were compared with the results of an *ex vivo* study with ^223^Ra.

**Results:**

The d_Lymph_ values differed from the d_Blood_ values by −1.0% (^90^Y), −5.2% (^99m^Tc), −22.3% (^123^I), 0.35% (^131^I), 2.4% (^177^Lu), −5.6% (^223^Ra) and −6.1% (^225^Ac). The number of DSB∙cell^−1^∙mGy^−1^ for each radionuclide was 0.014 DSB∙cell^−1^∙mGy^−1^ (^90^Y), 0.016 DSB∙cell^−1^∙mGy^−1^ (^99m^Tc), 0.013 DSB∙cell^−1^∙mGy^−1^ (^123^I), 0.012 DSB∙cell^−1^∙mGy^−1^ (^131^I), and 0.012 DSB∙cell^−1^∙mGy^−1^ (^177^Lu). These values agree very well with experimental data. The number of α-tracks∙100 cells^−1^∙mGy^−1^ for ^223^Ra and ^225^Ac where 0.144 α-tracks∙100 cells^−1^∙mGy^−1^ and 0.151 α-tracks∙100 cells^−1^∙mGy^−1^, respectively. These values agree very well with experimental data. Moreover, the linear density of DSBs per micrometer *α-track* length were 11.13 ± 0.04 DSB/µm and 10.86 ± 0.06 DSB/µm for ^223^Ra and ^225^Ac, respectively.

**Conclusion:**

This study describes a model to simulate the DNA DSB damage in lymphocyte nuclei validated by experimental data obtained from internal *ex vivo* blood irradiation with radionuclides frequently used in diagnostic and therapeutic procedures in nuclear medicine.

## Introduction

The simulation of DNA damage using Monte Carlo track structure codes has proven to help expand the understanding of how ionizing radiation (IR) interacts with the DNA, specifically in simulating DNA single- and double-strand breaks (SSBs and DSBs) [1], [2]. The main advantage of Monte Carlo track structure simulations is registering the spatial distribution of interactions of IR with DNA and quantifying the amount of deposited energy in each interaction [3]. The simulation of radiation-induced DNA SSBs and DSBs using Monte Carlo track structure codes is performed mainly in two different techniques: 1) By the interaction of IR with the three-dimensional structure of a DNA molecule [3] or by using clustering algorithms based on the identification of DNA strand breaks according to the spatial distribution of interactions of IR with a water medium [4], [5]. In both cases, it is necessary to apply predefined parameters (e.g., deposited energy thresholds and distance between interactions) to assess the DNA damage probability [6]. Moreover, other processes (e.g., physicochemical and chemical) can be considered after the interactions of IR with a cellular nucleus.

Beyond the Monte Carlo simulation, validating the simulation model with experimental data is fundamental [1]. The response of the DNA molecule to the IR-induced damage, specifically the production of γ-H2AX and 53BP1 radiation-induced foci (RIF) indicative of DSBs [7], is the counterpart to Monte Carlo simulated DNA DSB [2]. For an ideal validation of the Monte Carlo Simulation a 1:1 correspondence between RIF and DSB is needed. This condition is partially achieved at low radiation absorbed doses, where RIF saturation (number of RIF is smaller than the number of DSBs) is less likely to occur [8]. Moreover, various factors such as cellular repair mechanisms, RIF detectability at early time points after irradiation, accessibility of repair proteins at the DSB site, and the bystander effect can still affect and may alter correspondence of RIF and DSB [7].

The present study describes a method for validating a Monte Carlo track structure model based on a clustering algorithm that quantifies the number of DSBs produced in the lymphocyte nucleus after internal *ex vivo* irradiation of whole blood in a test tube with ^90^Y, ^99m^Tc, ^123^I, ^131^I, and ^177^Lu using GATE [9], [10] at the macroscopic level and Geant4-DNA [11], [12], [13] codes at the microscopic level. The validation is performed with experimental data in the range of low absorbed doses to the blood frequently encountered in nuclear medicine settings [14], [15], [16], [17], [18]. Moreover, a cluster analysis is implemented to establish the first data set of the number of clusters vs. cluster size (number of strand breaks) for internal *ex vivo* irradiation with radionuclides used in nuclear medicine.

## Materials and methods

This study simulates the blood irradiation experiments performed by Eberlein *et al.* [14], Schumann [17], and Göring *et al.* [15]. A description of these experiments is provided in the Supplemental data.

**Figure 1:**
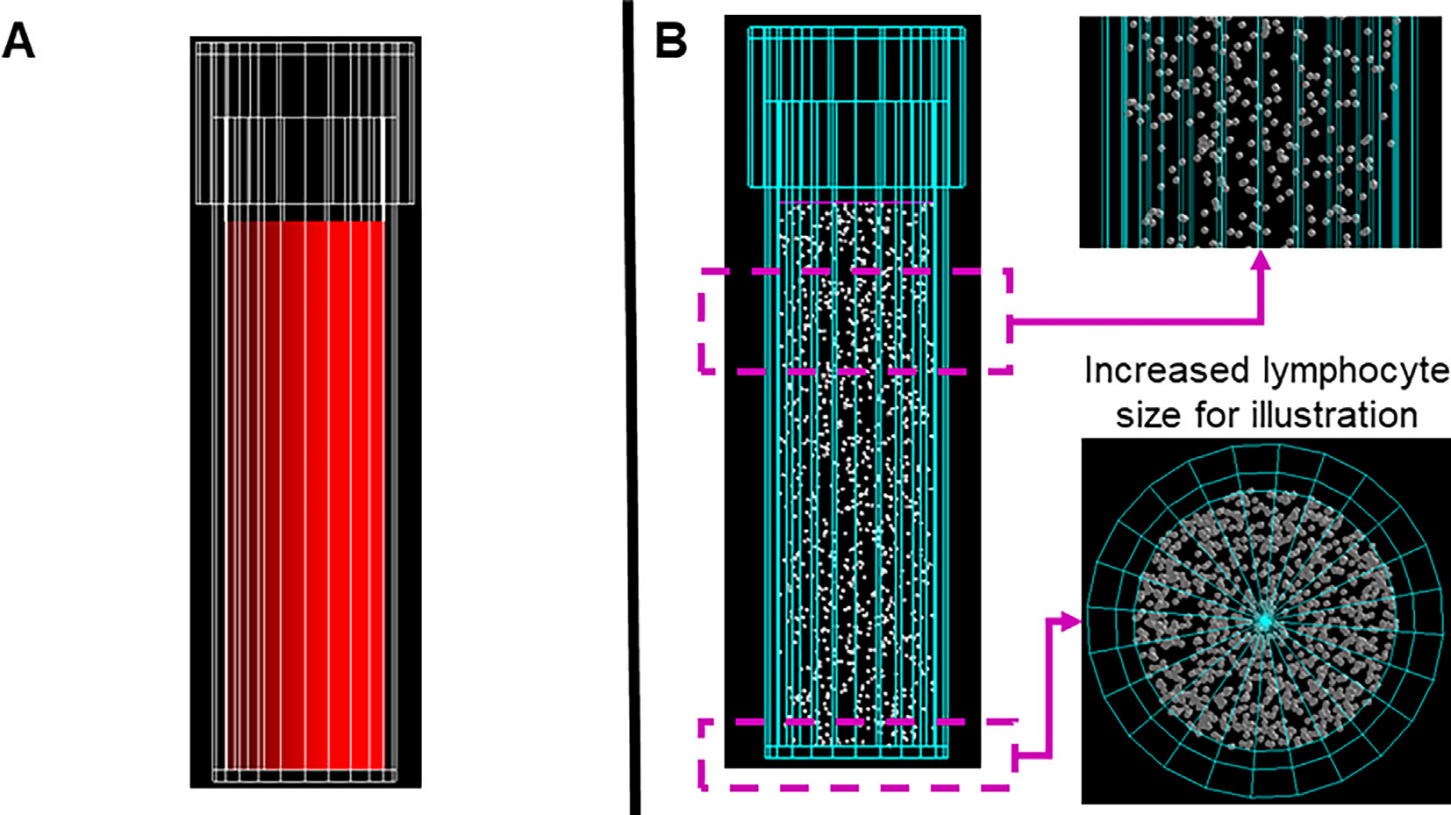
The layout of the simulation space in GATE: A) Macroscopic geometry: whole blood (8 mL vial) [19]. B) Macroscopic geometry, including the lymphocyte model.

This study is based on two simulation steps. First, a radiation transport at a macroscopic level using GATE was performed. The simulation geometry reproduces the irradiation geometry of a blood sample in an 8 mL vial with a screw cap (Sarstedt, No. 60.542.007, Germany) (Fig. 1A). Seven different radionuclide sources (^90^Y, ^99m^Tc, ^123^I, ^131^I, ^177^Lu, ^223^Ra, and ^225^Ac) were simulated. A detailed description of the simulation at the macroscopic level has been documented previously [19]. In comparison with our previous study, a geometric model of lymphocytes was added to the simulation.

The second step of this study consisted in quantifying the absorbed dose coefficients for lymphocytes’ nuclei per milliliter of blood (d_Lymph_) for 1 h of internal *ex vivo* irradiation of whole blood with different radionuclides and the number of radiation-induced DSBs, using Geant4-DNA, a setup that has been followed experimentally before [19].

The simulations were performed using GATE version 9.1 installed in combination with Geant4 version 10.7. Geant4-DNA simulations were also performed with the Geant4 version 10.7.

### Geometric model of lymphocytes

First, a lymphocyte was defined as a sphere with a radius of 3.75 μm and a nucleus of 3.1 μm. These values are mean values and were obtained from a specialized light microscopy experiment [20]. Next, using a python code, 1000 spheres of 3.75 μm radius [20] were used to mimic lymphocytes and distributed randomly inside of a cylindrical volume of 8 mL (0.72 mm radius and 49.12 mm height [19], avoiding overlapping (Fig. 1B). The coordinates of each sphere were recorded and stored in an external text file (TFile1). Moreover, a second text file (TFile2) with the corresponding GATE scripts to construct the 1000 spheres composed of water with their respective coordinates and spatial translations was generated. Each sphere was defined as an independent logic volume and randomly located inside of the 8 mL vial volume.

### Simulation at the macroscopic level using GATE

First, the *geometric model of lymphocytes* was included in the GATE geometry using the TFile2. The 1000 spheres were defined as cold sources (primary events inside of the spheres were forbidden), and a phase space actor was attached to each sphere [19] (the task of the phase space actor is to record the type, energy, direction, and hit position of each particle that hit the surface of each of the 1000 spheres). The number of primary events (nuclear transformations, nt) in GATE simulation varies according to the radionuclide to achieve a relative uncertainty of less than 5% in the mean deposited energy of the lymphocyte nucleus in the Monte Carlo simulation at the microscopic level (*E_Deposited,Lymph_*): 2 × 10^9^ nt for ^90^Y, 8 × 10^9^ nt for ^99m^Tc, 8 × 10^9^ nt for ^123^I, 4 × 10^9^ nt for ^131^I, 4 × 10^9^ nt for ^177^Lu, 2 × 10^9^ nt for ^223^Ra, and 2 × 10^9^ nt for ^225^Ac. The respective output of this first simulation was the phase spaces of the 1000 lymphocytes (1000 spheres of 3.75 μm radius).

### Simulation at the microscopic level using Geant4-DNA

**Figure 2:**
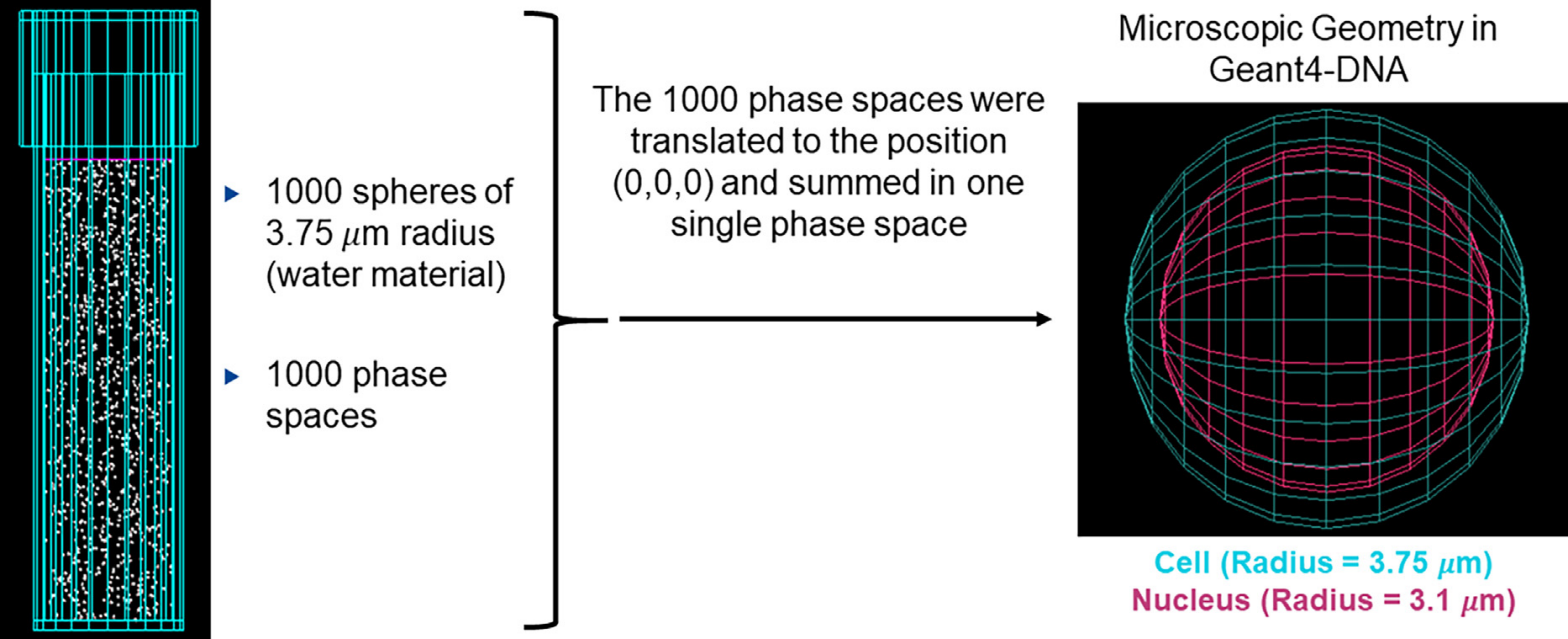
The layout of the simulation space: From macroscopic geometry (GATE) to microscopic geometry (cellular level) in Geant4-DNA.

The 1000 phase spaces were analyzed with a python code. First, each phase space was read and transformed into a database. Next, the coordinate system of each phase space was translated to the origin ((x,y,z) = (0,0,0)) using the coordinates file generated with the geometric model of lymphocytes (Fig. 2). Subsequently, all the databases were summed and converted into a single database (or *total phase space*). To reduce the size of the database, all the anti-neutrinos produced by the beta decay in all the simulated radionuclides were removed. Specific information for each particle in the database was selected (e.g., name, weight, kinetic energy, position, direction, event ID) and stored in a file *h5* (HDF5) format. Subsequently, the *total phase space* was used as a source by Geant4-DNA inside the *clustering* example [4]. To read the *h5* file and use it as a source, a new Geant4/C++ class was used. This C++ class was developed at the Laboratoire de Physique de Clermont (University Clermont Auvergne, France).

The simulation at the microscopic level does not consider radionuclide internalization or membrane binding. To our knowledge, there is no evidence of these scenarios in lymphocyte irradiation experiments with dissolved radionuclides. Moreover, in the experimental procedure (Supplemental data), the remaining activity after washing was always below 0.2%.

Lastly, to run the simulation, some changes in the de DetectorConstructor, RunAction, EventAction, and StepingAction classes of the *clustering* example were applied. These classes were added in the Supplemental file. The SteppingAction class was included for ^177^Lu and ^223^Ra to provide access to the implemented track length function for alpha particles.

#### DetectorConstruction class

The simulation space was composed of three volumes: 1) *World*: Sphere of 3.76 μm radius filled with the G4_Galactic material. The *World* excess in 0.01 μm the *Cell* radius to allow for phase space positioning. 2) *Cell*: Sphere of 3.75 μm radius [20] located at the isocenter of the *World* volume and filled with the G4_Water material. 3) *Nucleus*: Sphere of 3.1 μm radius [20] located at the isocenter of the *Cell* volume and filled with the G4_Water material. Furthermore, the nucleus volume was defined as a scoring volume for calculating the deposited energy and the outputs of the clustering algorithm.

#### RunAction and EventAction class

To extract the results of the clustering algorithm as a database, the number of single-strand breaks (SSBs), complex SSBs (defined as 2 SSBs or more), DSBs, deposited energy per particle, absorbed dose per particle, and distribution of cluster sizes per particle were saved as tuples into a *root* file. This *root* file was read with a python code, which attached the outputs of the clustering algorithm to the *total phase space*. This allowed correlating the particle type with each of the results of the clustering algorithm. This last general database will be called the *complete database* to explain future data processing steps.

#### SteppingAction class

A C++ conditional operator that considers only the interaction inside the nucleus volume was inserted into the *SteppingAction class.* This restricted the calculations of the clustering algorithm and the scoring of the deposited energy to the nucleus volume.

Moreover, to calculate the length of the alpha track (^223^Ra and ^225^Ac), a conditional operator, which selects only alpha particles, was included in the C++ code. In addition, the length of each step was recorded, and a total alpha track length was calculated. The total alpha track length was added to the output *root* file.

#### Physics list

The physics list of the *dnaphysics* example [12], [13] was used instead of the physics list of the *clustering* example. The physics processes were simulated using the physics list G4EmDNAPhysics_option4 in combination with the G4EmDNAPhysicsActivator. This approach permitted radiation transport of electrons above 10 keV (upper limit of the physics list G4EmDNAPhysics_option4). For radionuclide beta and gamma emitter, the lowest limit of secondary electron production (G4ProductionCutsTable) was adjusted to obtain the lowest difference between the lymphocyte nucleus mean deposited energy (*E_Deposited,Lymph_*) and the deposited energy in the blood (*E_Deposited,Blood_*) (data taken from publication [19].

#### Scoring of deposited energy:

At the microscopic level (using Geant4-DNA), the total deposited energy in the nucleus per particle was scored inside the *clustering* example, extracted, and stored in the *complete database*. Using a python code, the energy deposited in the lymphocyte nucleus per nuclear transformation was calculated from the *total database* using the following definition:

(1) $$E_{\mathrm{Deposited},Lymph}\left[\frac{\mathrm{keV}}{\mathrm{nt}}\right]=\frac{\sum_{i}^{n}E_{i}}{N_{\mathrm{Cells}}∙nt}=\frac{\mathrm{totaldepositedenergy}}{N_{\mathrm{Cells}}∙nt}$$

nt(nuclear transformations) is the number of simulated events in the GATE simulation. $E_{i}$ is the energy deposited by the particle i of the *total phase space*. $N_{\mathrm{Cells}}$ is equal to 1000; it corresponds to the number of lymphocytes (spheres) in the vial. The uncertainty of E_Deposited,Lymph_ using a history-by-history method was calculated by the expression:

(2) $$S_{E_{\mathrm{Deposited},Lymph}}\left[\frac{\mathrm{keV}}{\mathrm{nt}}\right]=\frac{1}{N_{\mathrm{Cells}}}\sqrt{\frac{1}{N-1}\left(\frac{\sum_{i}^{n}E_{i}^{2}}{N}-{\left(\frac{\sum_{i}^{n}E_{i}}{N}\right)}^{2}\right)}$$

### Calculation of the dose coefficient for lymphocyte nucleus in 1 mL of blood (d_Lymph_) during 1 h of internal ex vivo irradiation of whole blood

The calculation of *d_Lymph_* followed the same approach as described previously [19], using a mass of 1.24·10^−13^ kg (mass of the nucleus). Lastly, the *d_Lymph_* were compared with whole blood absorbed dose coefficients (*d_Blood_*) [19].

### Clustering algorithm

The clustering example included in Geant4-DNA code is based on a density-based spatial clustering of applications with noise (DBSCAN) algorithm [4], which was parameterized to simulate the DNA as a water-equivalent unstructured and randomly distributed sensitive volume of 7% volume fraction (*SPointsProb*: volume occupied by the DNA or sensitive volume in the lymphocyte nucleus). In this study, the 7% *SPointsProb* value is obtained by the ratio of the DNA volume of 8.3 µm^3^ (cylinder of 1.1 × 10^−9^ m radius [21] and a length of 2.2 m (0.34 × 10^−9^ m/bp [21] × 6.4 × 10^9^ bp [22] and the volume of the lymphocyte nucleus of 124.8 µm^3^ (sphere of 3.1 × 10^−6^ m radius). A single-strand break (SSB) is defined as a particle interaction inside of the 7% DNA volume fraction with 0% damage probability for energies below 5 eV (*EMinDamage*) increasing linearly to 100% at 37.5 eV (*EMaxDamage*) and above. The *damage probability* [4] function in the clustering example follows the next selection criteria:

(3) $$\mathrm{Damageprobability}\left(0<X\leq 1\right)=\frac{E_{\mathrm{Deposited},particle}-EMinDamage}{\mathrm{EMaxDamage}-EMinDamage}$$

here $E_{\mathrm{Deposited},particle}$ is the energy deposited by a particle inside of the sensitive volume. An interaction is included in the algorithm if X>a, where a is a randomly generated number from a uniform distribution.

Lastly, a DSB is defined as two (MinPts) SSBs separated by less than 10 base pairs (∼3.4 nm).

### Quantification of the number of DSBs

The total number of DSBs was extracted from the *complete database* using a python code to correlate the number of DSBs with the number of radiation-induced foci per cell and absorbed dose (*RIF_Exp_(RIF∙cell^−1^∙mGy^−1^)*) provided by experimental data. First, the activity associate to the number of simulated events (*nt*) was obtained from the equation:

(4) $$A\left[MBq\right]=\frac{\mathrm{nt}}{N\left(1h\right)\left[\frac{\mathrm{nt}}{\mathrm{Bq}}\right]∙10^{6}\left[\frac{\mathrm{Bq}}{\mathrm{MBq}}\right]}$$

Here $N\left(1h\right)$ is the number of nuclear transformations occurring during 1 h of blood irradiation [19].

Second, the absorbed dose to the whole blood (*D_Absorbed,Blood_*) was calculated using the absorbed dose coefficient (*d_Blood_*) from [19]:

(5) $$D_{\mathrm{Absorbed},Blood}\left[mGy\right]=\frac{A\left[MBq\right]∙d_{\mathrm{Blood}}\left[\frac{\mathrm{mGy}∙mL}{\mathrm{MBq}}\right]}{8[mL]}$$

Here, $d_{\mathrm{Blood}}$ is the whole blood absorbed dose coefficient in an 8 mL vial (water-equivalent medium).

Lastly, to obtain the number of DSB per cell and absorbed dose (*DSB_MC_(DSB∙cell^−1^∙mGy^−1^)*), the number of DSB per cell is divided by the absorbed dose to the whole blood:

(6) $${\mathrm{DSB}}_{\mathrm{MC}}=\frac{\mathrm{DSB}}{N_{\mathrm{Cells}}∙D_{\mathrm{Absorbed},Blood}\left[mGy\right]}$$

Furthermore, the uncertainty associated with *DSB_MC_* was calculated by the standard deviation of five Geant4-DNA simulations with different seeds. Lastly, the simulated data (*DSB_MC_*) was compared with experimental data (*RIF_Exp_*) from previous publications [14], [17].

### Quantification of the number of alpha tracks (α-track)

Experimentally, the visually counted number of α-tracks is used as a radiobiological marker for alpha emitting radionuclides [15], [16], [23]. Therefore, the total number of alpha particles and their track length were extracted from the *complete database*. An equivalent procedure to the DSBs calculation (section 6) was applied to determine the number of α-track per cell and absorbed dose (*α-track_MC_(α-track ∙100 cells^−1^∙mGy^−1^)*). Furthermore, the uncertainty associated with *α-track_MC_* was calculated by the standard deviation of five Geant4-DNA simulations with different seeds. Additionally, a second value of the number of *α-track_MC,Treshold_(α-track∙100 cells^−1^∙mGy^−1^),* was calculated using experimental-based thresholds (*α-tracks* with a track length superior to 0.75 μm and more than 7 DSBs). Considering a resolution limit of 250–300 nm for a RIF, an alpha track must have at least three adjacent foci in a row. Therefore, the shortest tracks should be between 0.75–0.9 µm. For validation, the simulated data (*α-track_MC_*) with and without threshold were compared with experimental data (*α-track_Exp_*) from a previous publication of our group [15].

Lastly, the linear density of DSBs per α-track length (*DSB_MC,α-track_(DSB∙µm^−1^)*) was calculated by considering the total number of DSBs per cell and the α-track length per cell (information extracted from the *.root* data file. Section: 3.3. SteppingAction class).

### Cluster analysis

The number of clusters and the cluster size (number of strand breaks) for each particle in the phase space were extracted from the *clustering* example and integrated into the *complete database*. This step made it possible to organize the number of clusters per cell and absorbed dose classified by the number of strand breaks in a histogram. Moreover, by selecting only the particles that produced DSBs (number of DSBs larger than 0 DSBs), a histogram of the number of clusters per cell and absorbed dose with simple DSBs and complex DSBs (defined as 3 SSBs or more, with at least 1 SSB on the opposite chain) was generated.

## Results

### Comparison between the dose coefficient for lymphocyte nucleus and dose coefficient for whole blood

**Figure 3:**
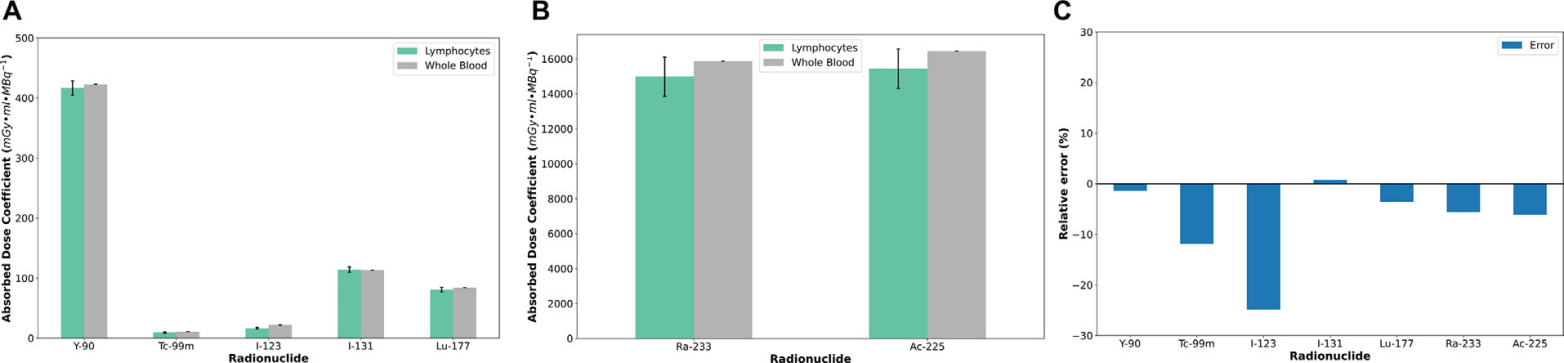
Absorbed dose coefficients for lymphocytes (*d_Lymph_*) using Geant4-DNA and whole blood (*d_Blood_*) using GATE [19]: A) Values for beta- and gamma-emitters. B) Values for alpha emitters. C) Relative error between *d_Lymph_* and *d_Blood_* (reference: *d_Blood_*).

This section presents a comparison between the dose coefficient for lymphocyte nucleus in 1 mL of blood (*d_Lymph_*) under 1 h of internal *ex vivo* irradiation of whole blood and the dose coefficient for whole blood in 1 mL of blood (*d_Blood_*) under 1 h of internal *ex vivo* irradiation reported in a previous publication [19]. Fig. 3 shows the respective dose coefficients for the simulated radionuclide: Fig. 3A presents a plot with the value for beta and gamma emitters, and Fig. 3B for alpha emitters. The *d_Lymph_* values are reported in Table 1 of the Supplemental material. In the case of beta and gamma emitters, the relatives errors between *d_Lymph_* and *d_Blood_* ranged from −24.8% for ^123^I to 0.8% for ^131^I, while for alpha emitters, the relative errors were −5.6% for ^223^Ra and −6.1% for ^225^Ac. All the relative errors are plotted in Fig. 3C. For radionuclide beta and gamma emitters, the lowest limit for secondary electron production (G4ProductionCutsTable) were 105 eV for ^99m^Tc, 79 eV for ^123^I, 105 eV for ^131^I, 105 eV for ^177^Lu, and 350 eV for ^90^Y.

Lastly, the electron, gamma, and alpha input spectra (particles in the phase space) for each radionuclide and a plot of energy deposition vs. kinetic energy are included in Figs. 1–7 of the Supplemental material.

### Comparison between the number of DSBs and RIF for radionuclide beta and gamma emitters.

**Figure 4:**
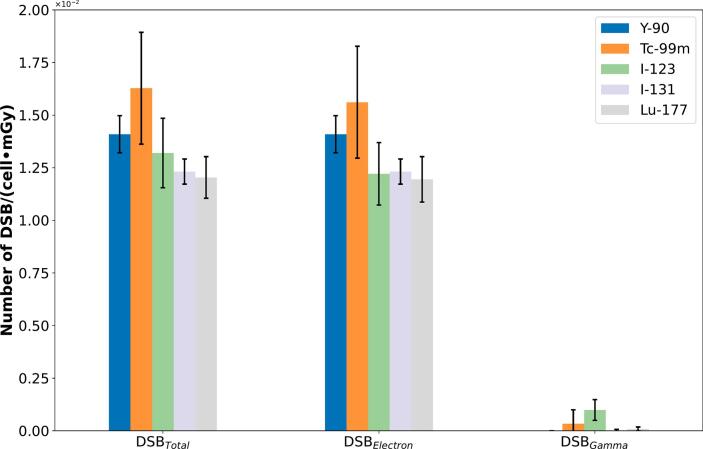
Total number of DSBs for beta- and gamma-emitter and their contributions per particle (electrons or gammas). Error bars consider a coverage factor (k) of 2.

**Figure 5:**
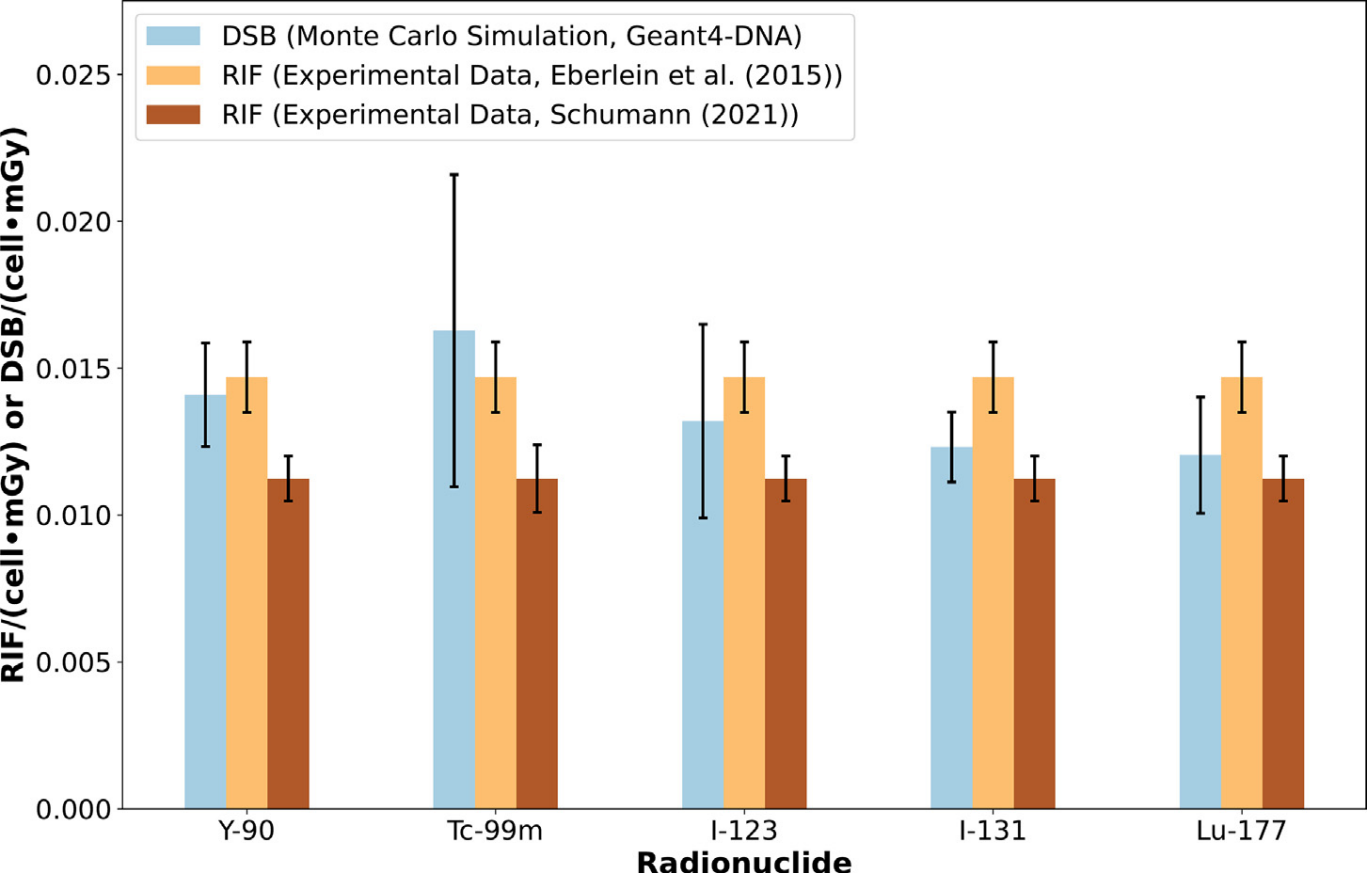
Comparison between the Monte Carlo-based total number of *DSB_MC_* for beta- and gamma-emitters and number of radio-induced foci (*RIF_Exp_*) per cell measured experimentally [14], [15]. Error bars consider a coverage factor (k) of 2.

Fig. 4 illustrates the number of DSB per cell and absorbed dose (*DSB_MC_*) obtained from the Geant4-DNA simulation, where the total value is composed by the DSBs produced by electron and gamma particles. Moreover, the number of *DSB_MC_* stays between 0.016 ± 0.003 DSB∙cell^–1^∙mGy^–1^ (k = 1) for ^99m^Tc and 0.012 ± 0.001 DSB∙cell^–1^∙mGy^–1^ (k = 1) for ^177^Lu. Lastly, the comparison of our simulation model against experimental data is illustrated in Fig. 5 (*DSB_MC_* vs. *RIF_Exp_*). Two experimental data sets are used for comparison: 1) Eberlein *et al.* [14]: 0.0147 RIF∙cell^−1^∙mGy^−1^ (irradiation of blood with ^177^Lu and ^131^I) and 2) Schumann [17]: 0.0117 RIF∙cell^−1^∙mGy^−1^ (irradiation of blood with ^177^Lu, ^90^Y, ^68^Ga und ^99m^Tc). Schumann’s study used a larger number of subjects and radionuclides than Eberlein’s study. Moreover, Schumann’s study used absorbed dose coefficients calculated for a blood vessel geometry [24], while Eberlein *et al.* [14] used absorbed dose coefficients calculated for the vial geometry used in the irradiation experiments.

### Range of absorbed doses in blood reached in the simulation

The activities associated with the number of simulated primary particles (equation (4)) are 0.56 MBq for ^90^Y, 2.4 MBq for ^99m^Tc, 6.1 MBq for ^123^I, 2.3 MBq for ^131^I, 1.1 MBq for ^177^Lu, 0.3 MBq for ^223^Ra, and 0.3 MBq for ^225^Ac.

Using equation (5), the *D_Absorbed,Blood_* for each radionuclide and its respective number of simulated primary particles (*nt)* was calculated. The values for radionuclides with beta and gamma emission are in the range of low absorbed doses (<100 mGy): 28.4 mGy for ^90^Y, 3.0 mGy for ^99m^Tc, 6.1 mGy for ^123^I, 15.1 mGy for ^131^I, and 11.1 mGy for ^177^Lu.

For ^223^Ra and ^225^Ac the absorbed doses were 538 mGy and 544 mGy. Experimentally in alpha emitting radionuclides, the number of alpha tracks is used to quantify the DNA damage. Therefore, a high absorbed dose increased the number of possible alpha tracks improving the statistics of the simulation (observed on the error bars of Fig. 7).

### Cluster analysis for radionuclide beta and gamma emitters

**Figure 6:**
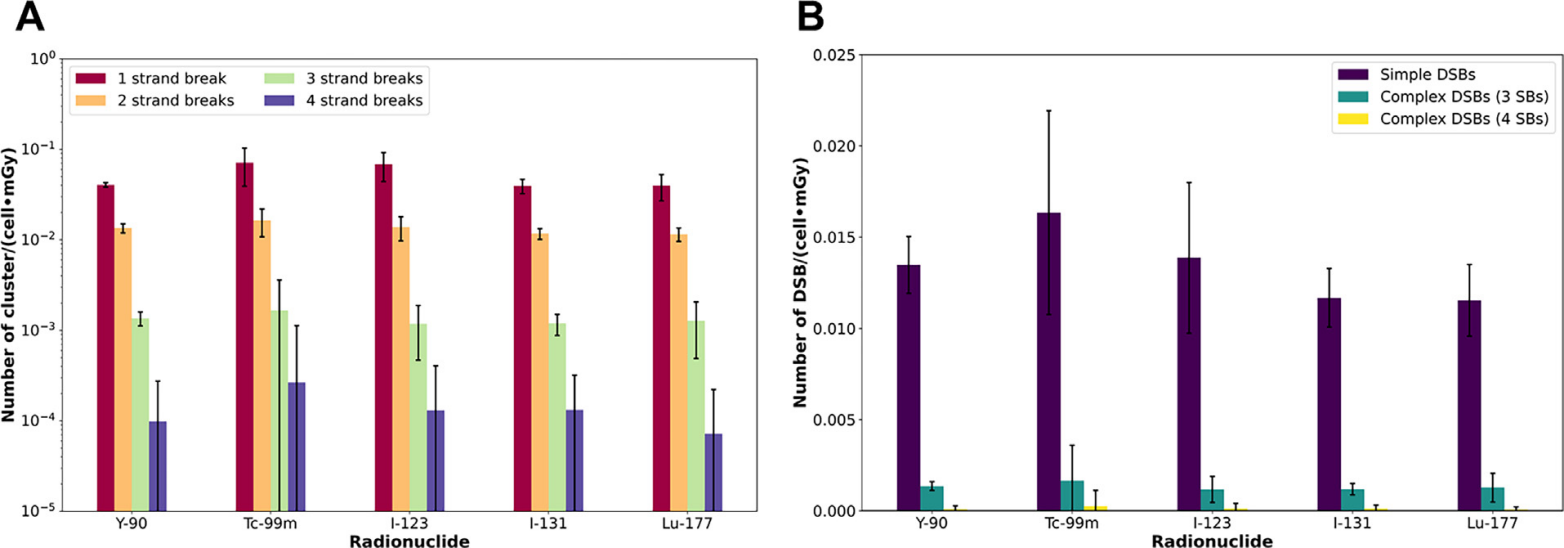
Cluster analysis (complexity of the DNA damage) for beta- and gamma-emitters: A) Classification of the cluster based on the number of strand breaks (SBs). B) Simple and complex DSBs. Error bars consider a coverage factor (k) of 2.

Fig. 6A shows the number of clusters per cell and absorbed dose classified by the number of strand breaks, where only clusters with 1 strand break are certain to be single strand breaks (SSBs), while clusters with 2 strand breaks can be clusters with 2 SSBs in opposite strands (simple DSB) or clusters with 2 SSBs in the same strand. In clusters with 3 and 4 strand breaks, many DNA damage arrangements are possible (e.g., 3 strand breaks → 1) a complex DSB: 2 SSBs in one strand and 1 SSB in the opposite strand, 2) 3 SSBs in one strand. 4 strand breaks → 1) a complex DSB: 2 SSBs in one strand and 2 SSBs in the opposite strand, 2) 4 SSBs in one strand). Fig. 6B shows only the number of clusters with simple DSBs and complex DSBs per cell and absorbed dose.

### Comparison between α-track_MC_ and α-track_Exp_

**Figure 7:**
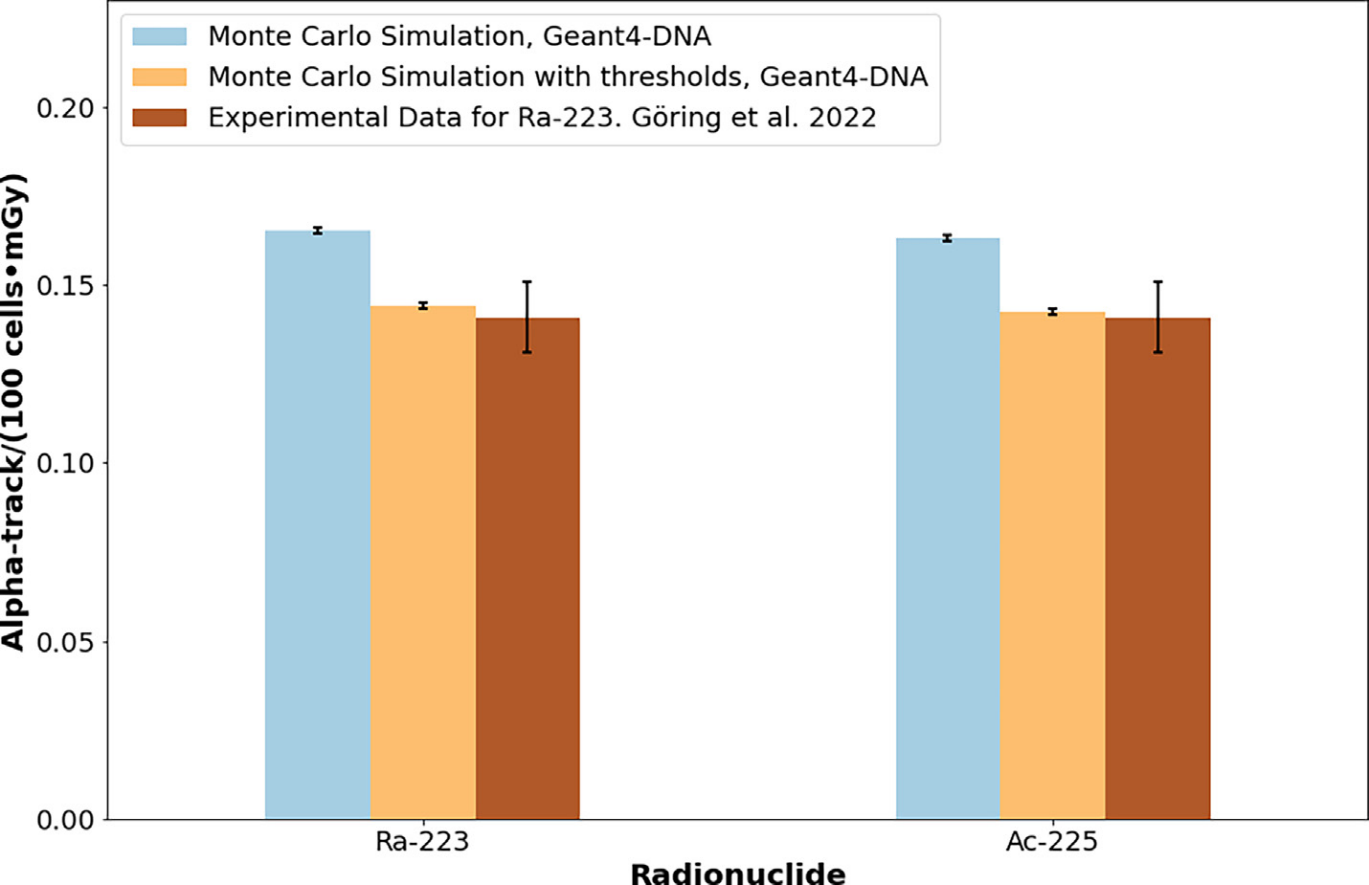
Comparison between the Monte Carlo-based total number of *α-track_MC_* for alpha-emitters and the number of *α-track_Exp_* measured experimentally [15]. Error bars consider a coverage factor k = 2.

Fig. 7 shows the number of α-tracks per 100 cells and absorbed dose (*α-track_MC_*) obtained from the Monte Carlo simulation in Geant4-DNA and experimentally [15]. Two values were obtained from the simulation: 1) Monte Carlo simulation total number of *α-track_MC_*: 0.1654 ± 0.0008 *α-track·100 cell^−1^·mGy^−1^ for* ^223^Ra and 0.1634 ± 0.0008 *α-track·100 cell^−1^·mGy^−1^ for* ^225^Ac. 2) Monte Carlo simulation with thresholds(*α-track_MC,Threshold_: α-track_MC_* larger than 0.75 µm in length and with more than 7 DSBs): 0.1654 ± 0.0008 *α-track·100 cell^−1^·mGy^−1^ for* ^223^Ra and 0.1634 ± 0.0008 *α-track·100 cell^−1^·mGy^−1^*. The relative error between the Monte Carlo simulation (total number of *α-tracks*) and the experimental data for ^223^Ra [15] is 17.3% for ^223^Ra, while the relative error between the Monte Carlo simulations with thresholds and the experimental data for ^223^Ra [15] is 2.7%.

### Cluster analysis for radionuclide alpha emitters

**Figure 8:**
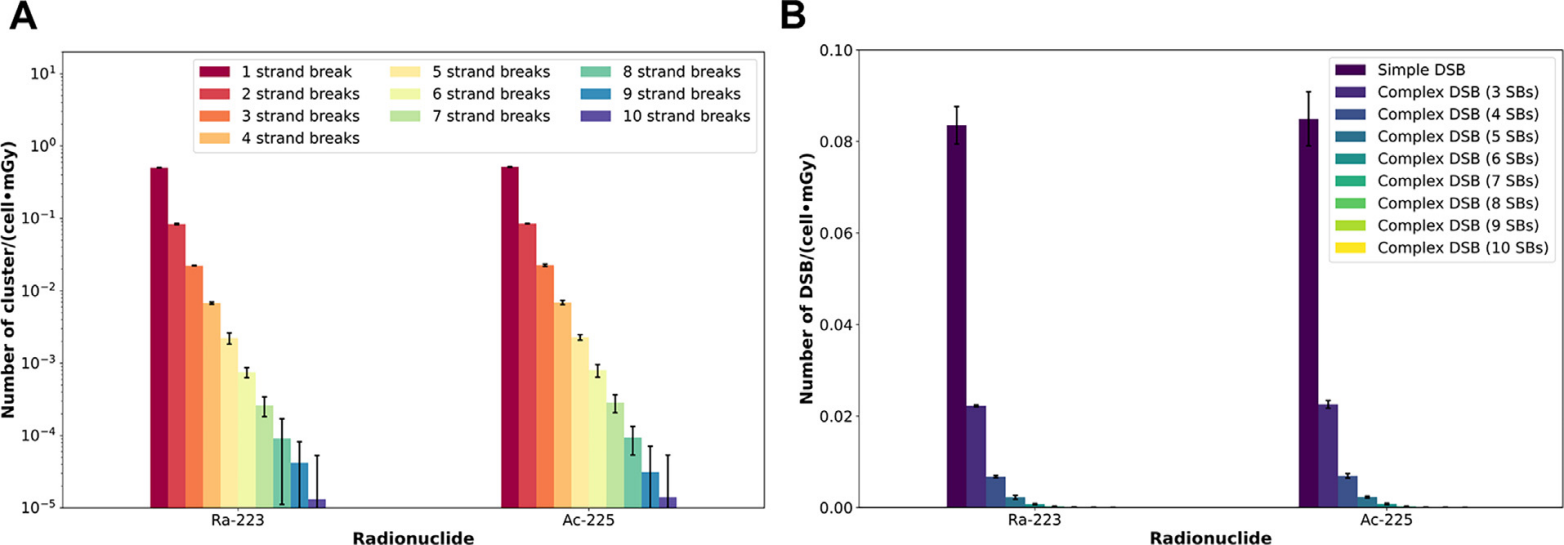
Cluster analysis (complexity of the DNA damage) for alpha-emitters: A) Classification of the cluster based on the number of strand breaks (SBs). B) Simple and complex DSBs.

Fig. 8A shows the number of clusters per cell and absorbed dose classified by the number of strand breaks as a parameter. Fig. 8B shows only the number of clusters per cell and absorbed dose with simple DSBs and complex DSBs. Furthermore, the linear density of DSBs per µm *α-track* length (*DSB_MC,α-track_(DSB∙µm^−1^)*) were 11.13 ± 0.04 DSB/µm and 10.86 ± 0.06 DSB/µm for ^223^Ra and ^225^Ac, respectively, aligning with experimental data for ^223^Ra [25].

## Discussion

Data comparing results of Monte-Carlo simulations of DNA damage to experimental data are sparse and restricted to data obtained from experiments using X-ray irradiation [26] or ion microbeam irradiation of primary human endothelial cell cultures with alpha particles or protons [27].

In this study, we validated a DNA damage model for nuclei of human lymphocytes internally irradiated with radionuclides used in nuclear medicine at low absorbed doses (<100 mGy), occurring in blood during molecular radiotherapies [28], [29], by using the clustering example included in the Geant4-DNA simulation tool kit [4], [13]. This example is based on a clustering algorithm for grouping DNA strand breaks produced in a liquid water geometry. The validation was performed by comparing the results of the Monte Carlo simulation against actual experimental data [14], [15], [17].

In the first section of this study, we compare the absorbed dose coefficients for the lymphocyte nucleus (*d_Lymph_*) (microscopic level) and whole blood (*d_Blood_*) (macroscopic level). Considering that the cell is defined as a cold source (without a self-irradiation component), the negative values observed in Fig. 3C are expected. The simulations of ^99m^Tc and ^123^I sources presented the largest relative error. These differences might be related to the high gamma emission probability of these radionuclides and the low interaction probability of gamma particles with the water medium. This results in ^99m^Tc and ^123^I sources having the largest number of DSBs caused by gamma particles (see Fig. 4C) in comparison with ^90^Y, ^131^I, and ^177^Lu sources. The only parameter in the Geant4-DNA simulation that permits adjusting the deposited energy (therefore, the absorbed dose coefficients) is the low energy limit of the production of secondary electrons (production thresholds in the *G4ProductionCutsTable*). Based on our user experience, this threshold directly impacts the energy deposition and DSBs production, for which both parameters follow a direct proportionality. In this study, the production threshold was optimized to reach the lowest difference between the absorbed dose coefficients for the lymphocyte nucleus (*d_Lymph_*) (microscopic level) and whole blood (*d_Blood_*) (macroscopic level), always favoring a negative difference.

In Fig. 3 we show that the number of *DSB_MC_* for all the beta and gamma emitter radionuclides are in a similar range independently of the differences in the emission probabilities (e.g., ^99m^Tc (140 keV, gamma) vs. ^90^Y (2.28 MeV, higher energy beta emissions)) [30]. Experimentally, Schumann [17] reported non-significant differences in the induction of RIF when considering the type of radionuclide or the individual subject for irradiation of blood with different radionuclides ^177^Lu, ^90^Y, ^68^Ga und ^99m^Tc in a group of four subjects and 16 blood samples.

The comparison of DSB_MC_ and RIF_Exp_ (Fig. 5) reveals that the DSBs calculated by simulation fall within the range of the values of two experimental data sets [14], [17] (assuming a 1:1 correspondence between RIF and DSB at a low absorbed dose range). The clustering example (based on a DBSCAN algorithm) estimates the DSBs using probabilistic criteria (*SPointsProb* and *damage probability*). The *SPointsProb* defines a uniform probability that a particle interaction is located inside a sensitive volume where this interaction can directly or indirectly reach the DNA [4]. The *SPointsProb* is specific for the simulation geometry and should be selected to meet the experimental results. However, in this study, the *SPointsProb* was obtained under geometrical criteria (considering the DNA volume as a cylinder of 1.1 × 10^−9^ m radius [21] and a length of 2.2 m [21] and dividing the volume of this cylinder by the volume of the lymphocyte nucleus). Piovesan *et al.* [31] estimated the mass of the human male and female genomes at 6.41 g and 6.51 g, respectively. These mass values led to a volume of 3.8 mL (using a DNA mass density of 1.7 g/mL [32] for the human male and female genomes. A 3.8 mL DNA volume resulted in a *SPointsProb* of 3%. This molecular-based value differs by 4% from the value used in this study. Francis *et al.* [4] defined the sensitive volume in a DBSCAN algorithm as the volume occupied by the DNA double helix plus a virtual aura surrounding the DNA. Bertolet *et al.* [33] demonstrated that Monte Carlo simulations of DNA damage encompass different volume models and specific parameters, which must be selected to meet experimental results. Therefore, the difference between a cylindrical- and molecular-based *SPointsProb* is necessary to consistently match the simulation with the experimental data for lymphocytes *ex vivo* irradiated with gamma-, beta-, and alpha-emitting radionuclides.

The complexity of the DNA damage was approached with the experiments underlying Fig. 6 by quantifying the number of simple and complex DSBs [34]. The number of DSBs calculated by Monte Carlo simulation is for the different gamma and beta emitters within the same range. In Fig. 5, as in Fig. 6B, it is shown that the number of DSBs for the ^99m^Tc and ^123^I radionuclides presented the largest error bars, which agrees with the error bars of the absorbed dose coefficients at the microscopic level in Fig. 3A.

The Monte Carlo simulation (without experimental thresholds) provides a larger number per 100 cells of *α-tracks* than the experimental data (Fig. 7). These differences might be related to the resolution limit of standard fluorescence microscopy (0.25–3 µm) [35]. Experimentally, to define an alpha track one needs at least three adjacent foci in a row [25], which corresponds to a shortest track of 0.75–0.9 µm. Moreover, experimentally only the roughly parallel traversing *α-tracks* can be easily identified. This study proposes experimental-based thresholds (*α-tracks* larger than 0.75 µm and with more than 7 DSBs [35], which show good agreement between the Monte Carlo-based *α-tracks* values and the experimental data [18].

The complexity of the DNA damage produced by alpha particles (Fig. 8) is higher than that produced by beta and gamma emitters (Fig. 6) [34], [36]. This complexity makes it impracticable to establish a relationship between DSBs and RIF. Therefore, the linear density of DSBs per micrometer *α-track* length (*DSB_MC,α-track_*) is used to assess the DNA damage produced by alpha particles. In this study, we calculate *DSB_MC,α-track_* values of 11.13 ± 0.04 DSB/µm and 10.86 ± 0.06 DSB/µm for ^223^Ra and ^225^Ac, respectively. Du *et al.* [23], in a cell irradiation experiment with 55 MeV carbon ions, quantify a *DSB_MC,α-track_* of 15 DSB/μm using the PARTRAC code [37]. In our study, the kinetic energy of alpha particles registered in the phase space stays between 0.01 MeV and 8.36 MeV for ^223^Ra and 0.01 MeV and 8.36 MeV for ^225^Ac. Therefore, considering the energy and particle mass differences our values are in a similar range than Du *et al.* [23]. In addition, Scherthan *et al.* [25] investigated the DNA damage caused by α-tracks of ^223^Ra in-solution irradiated leukocytes using super-resolution light microscopy (SMLM) with cluster analysis of single molecule signal-point density regions to observe the nanostructures of DNA damage α-tracks. They reported that in average 12 DSBs may be contained in the damaged area of an average γ-H2AX alpha particle track of 1.27 µm^2^ (diameter of a circular area = 1.27 µm) in a lymphocyte nucleus of 35 µm^2^ (in our study, the size of the nucleus used in the Monte Carlo simulation is 30.2 µm^2^). This results in a linear density of DSBs per micrometer of *α-track* length of 9.45 DSB/µm (12 DSB/1.27 µm). These data are in very good agreement with our simulation data.

### Comparison with previous X-ray irradiation studies

A first reference on the number of DSBs per cell per Gy in mammalian cells caused by low-LET radiation exposure is in Ward [38], who reported a value of 40 DSB·Cell^−1^·Gy^−1^. Most likely, this value is based on theoretical considerations and data obtained from electrophoretic DNA double-strand break assays. These assays are sensitive at absorbed doses of more than 1 Gy [39], a dose range not considered in this work. For lower absorbed doses, RIF, have been established as surrogate markers for DSBs [39].

Rothkamm *et al.* [40] reported a value of 36 RIF·cell^−1^·Gy^−1^ in primary human lung fibroblasts cells (MRC-5) in G1 phase under X-ray irradiation (90 kVp X-ray, dose rate of 2 Gy/min). Horn *et al.* [41] reported a value of 15.7 RIF·cell^−1^·Gy^−1^ for the irradiation of blood collected in EDTA vacutainer tubes using a 250 kVp X-ray source (dose rate of 1.7 Gy/min). As Barnard *et al.* [39] pointed out, an explanation of this difference could be that the absolute yield per unit absorbed dose changes linearly with the DNA content of the cells. Furthermore, the number of RIF/cell possibly depends substantially on the cell cycle [39], nuclear diameter and cell type.

For lymphocytes the value reported by Horn *et al.* is in agreement with our simulated data (assuming 1 RIF ≈ 1 DSB, between 12 DSB∙cell^−1^∙Gy^−1^ (^99m^Tc), and 16 DSB∙cell^−1^∙Gy^−1^ (^177^Lu)) and the experimental data used as a reference in this study (15 RIF∙cell^−1^∙Gy^−1^ [14] and 11 RIF∙cell^−1^∙Gy^−1^ [17].

### Study limitations

This Monte Carlo simulation model was validated against experimental data of lymphocytes irradiated with beta- and gamma-emitting radionuclides [14], [17] and ^223^Ra (alpha emitter) [15], [25] at low absorbed doses (<100 mGy). Therefore, this model is applicable to quantify the DNA damage in lymphocytes at low absorbed doses.

In this study, lymphocytes are modelled by spheres with a mean radius of 3.75 µm and nuclei with a radius of 3.1 µm. This approach has two advantages: 1) The irradiation profile of the lymphocytes’ considers the irradiation at several points inside the vial (centre and periphery). 2) A single simulation is required in Geant4-DNA using the sum of the 1000 phase spaces, which makes it possible to use a large number of particles, improve the statistics and optimize the simulation time at the cellular level. However, this approach does not represent the lymphocytes’ variation in size (e.g. Loiko *et al*. [20] found in a group of 16 individuals that the lymphocyte radius varied from 2.4 to 6.0 µm). Therefore, further studies will consider the uncertainty of lymphocytes’ size distribution.

Furthermore, further studies considering a three-dimensional DNA structure and indirect damage are necessary to assess individually all the components of the DNA and evaluate the impact of the spatial distribution of the DNA (e.g., helical periodicity of DNA on the nucleosome) damage in lymphocytes irradiated with radionuclides.

## Conclusion

This study validated a model to simulate the DNA damage for lymphocytes *ex vivo* irradiated with gamma, beta, and alpha-emitting radionuclides, assuming a 1:1 correspondence between RIF and DSB at a low absorbed dose range doses (<100 mGy). Moreover, it provides a methodology to reproduce the spatial distribution of lymphocytes in the volume of an irradiation vial and to determine the absorbed dose coefficient to lymphocytes in 1 mL of blood (*d_Lymph_*) for 1 h of internal *ex vivo* irradiation of whole blood, as applied in experimental settings [14], [15], [17]. In addition, this study provides a detailed methodology to couple a macroscopic model (vial) with a microscopic model (lymphocytes nucleus).

Geant4-DNA provides complementary information in assessing the DNA damage produced by alpha particles, explicitly quantifying the linear density of DSBs per micrometer along the length of an *α-track and the number of *simple and complex DSBs.

The sensitive volume (*SPointsProb* = 7%) used in this study provides a reliable approximation to assess the DNA damage that consistently matches the simulation data with the experimental data.

## Author contributions

All authors participated in the study design. Maikol Salas-Ramirez performed the methodology and calculations. All authors analyzed and interpreted the data and wrote the manuscript.

## Funding

This work was supported by a contract research project for the Bundeswehr Medical Service (Research grant number: E/U2AD/KA367/KF554) and in part by the 10.13039/501100001659German Research Foundation (Deutsche Forschungsgemeinschaft, Grant Number 509851852).

Maikol Salas-Ramirez was supported by funds of the Bavarian State Ministry of Science and Arts and the Graduate School of Life Sciences (GSLS) of the University of Würzburg.

The funders had no role in the study design, data collection and analysis, or manuscript preparation.

## Data Availability Statement

C++ and Python codes can be requested from the corresponding author.

## Declaration of Competing Interest

The authors declare the following financial interests/personal relationships which may be considered as potential competing interests: M. Lassmann has received institutional grants by IPSEN Pharma, Nordic Nanovector, and Novartis. No other potential conflicts of interest relevant to this article exist.
