# Supplementary material for: Radiation-induced double-strand breaks by internal ex vivo irradiation of lymphocytes: Validation of a Monte Carlo simulation model using GATE and Geant4-DNA
Source: Z Med Phys. 2023 Aug 18;35(3):235–47. doi: 10.1016/j.zemedi.2023.07.007 (PMC12664439; doi:10.1016/j.zemedi.2023.07.007)
Supplement: Supplementary Data 1 [file mmc1.pdf]

**Radiation-Induced Double-Strand Breaks by Internal Ex Vivo Irradiation of Lymphocytes: Validation of a Monte Carlo Simulation Model Using GATE and Geant4-DNA: Supplemental Material**

**Author Names:**

Maikol Salas-Ramirez[1]*, Lydia Maigne[2], Giovanna Fois[2], Harry Scherthan[3], Michael Lassmann[1], Uta Eberlein[1]

**Affiliations:**

[1]Department of Nuclear Medicine, University of Würzburg, Würzburg, Germany

[2]Laboratoire de Physique de Clermont, University of Clermont Auvergne, Clermont, France;

[3]Bundeswehr Institute of Radiobiology affiliated to the University of Ulm, Munich, Germany.

***Contact information:** Maikol Salas Ramirez, Department of Nuclear Medicine, University of Würzburg, Oberdürrbacher Str. 6, 97080 Würzburg, Germany, Phone: +49-931-201-35462

SUPPLEMENTAL TABLES

Supplemental table 1. Values of $d_{Lymph}$ for gamma, beta, and alpha emitters in water

| Radionuclide | $d_{Lymph}\left(\frac{mGy\text{-}ml}{MBq}\right)$ |
|---|---|
| $^{90}Y$ | 416.7 ± 5.9 |
| $^{99m}Tc$ | 9.4 ± 0.5 |
| $^{123}I$ | 16.6 ± 0.7 |
| $^{131}I$ | 114.3 ± 2.2 |
| $^{177}Lu$ | 81.1 ± 1.9 |
| $^{223}Ra$ | 14984.8 ± 558.5 |
| $^{225}Ac$ | 14969.6 ± 557.9 |

Supplemental table 2. Number of $DSB_{MC}$ obtained from the Monte Carlo simulation (Geant4-DNA) for beta- and gamma-emitters

| Radionuclide | $DSB_{MC}\left(\frac{DSB}{Cell \cdot mGy}\right)$ | | |
|---|---|---|---|
| | Total | Electron | Gamma |
| $^{90}Y$ | 0.0141 ± 0.0009 | 0.0141 ± 0.0009 | 0.0000 ± 0.0000 |
| $^{99m}Tc$ | 0.0163 ± ~~0.0266~~ | 0.0156 ± ~~0.0266~~ | 0.0003 ± 0.0007 |
| $^{123}I$ | 0.0132 ± 0.0016 | 0.0122 ± 0.0015 | 0.0010 ± 0.0005 |
| $^{131}I$ | 0.0123 ± 0.0006 | 0.0123 ± 0.0006 | 0.0000 ± 0.0001 |
| $^{177}Lu$ | 0.0120 ± 0.0010 | 0.0119 ± 0.0011 | 0.0001 ± 0.0001 |

Supplemental table 3. Number of α-tracks obtained from the Monte Carlo simulation (Geant4-DNA) for alpha emitters

| Radionuclide | $\alpha\text{-}track_{MC}\left(\frac{\alpha\text{-}track}{100\ cell \cdot mGy}\right)$ | |
|---|---|---|
| | Total | With experimental-based-thresholds* |
| $^{223}Ra$ | 0.1654 ± 0.0008 | 0.1442 ± 0.0009 |
| $^{225}Ac$ | 0.1634 ± 0.0008 | 0.1425± 0.0009 |

**α-tracks* larger than 0.75 μm and with more than 7 DSBs.

## SUPPLEMENTAL FIGURES

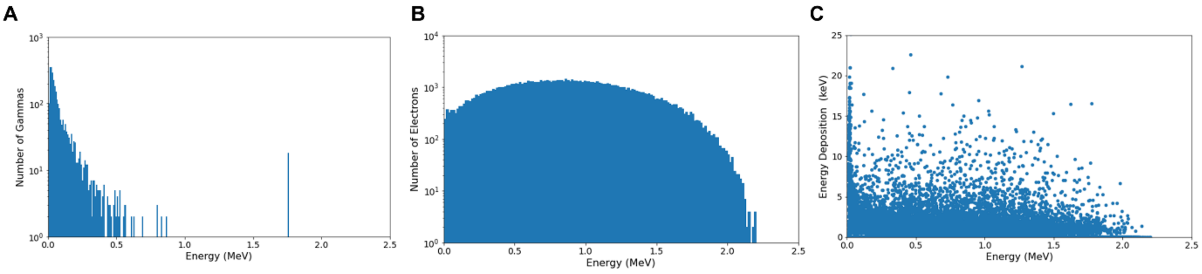


Supplemental figure 1. $^{90}Y$ phase space: A) Gamma spectrum in the phase space. B) Electron spectrum in the phase space. C) Energy deposition vs. energy for all particles in the phase space.

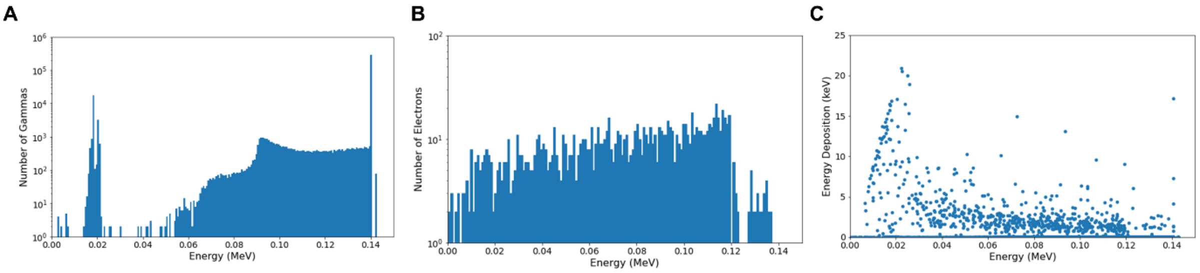


Supplemental figure 2. $^{99m}$Tc phase space: A) Gamma spectrum in the phase space. B) Electron spectrum in the phase space. C) Energy deposition vs. energy for all particles in the phase space.

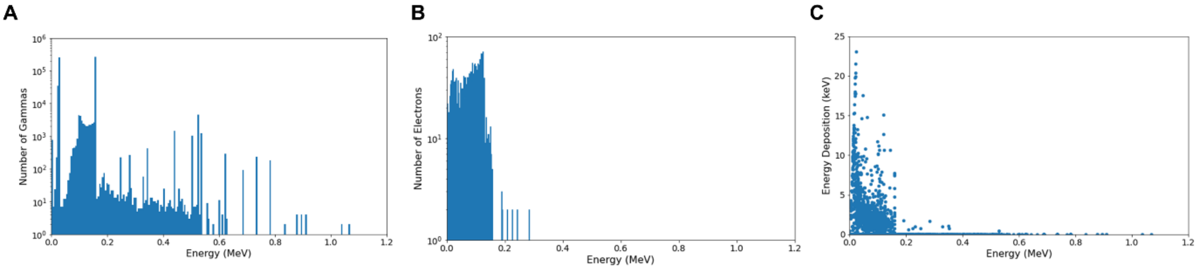


Supplemental figure 3. $^{123}$I phase space: A) Gamma spectrum in the phase space. B) Electron spectrum in the phase space. C) Energy deposition vs. energy for all particles in the phase space.

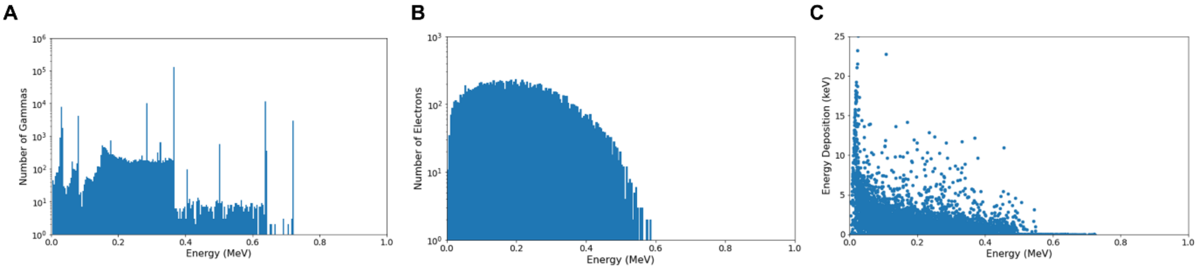


Supplemental figure 4. $^{131}$I phase space: A) Gamma spectrum in the phase space. B) Electron spectrum in the phase space. C) Energy deposition vs energy for all particles in the phase space.

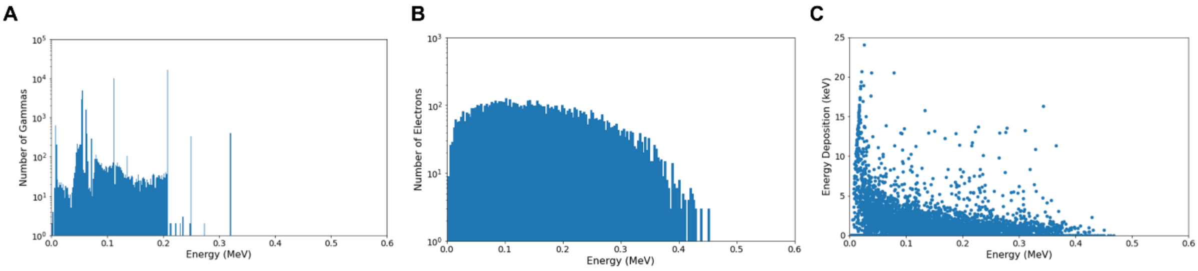


Supplemental figure 5. $^{177}$Lu phase space: A) Gamma spectrum in the phase space. B) Electron spectrum in the phase space. C) Energy deposition vs. energy for all particles in the phase space.

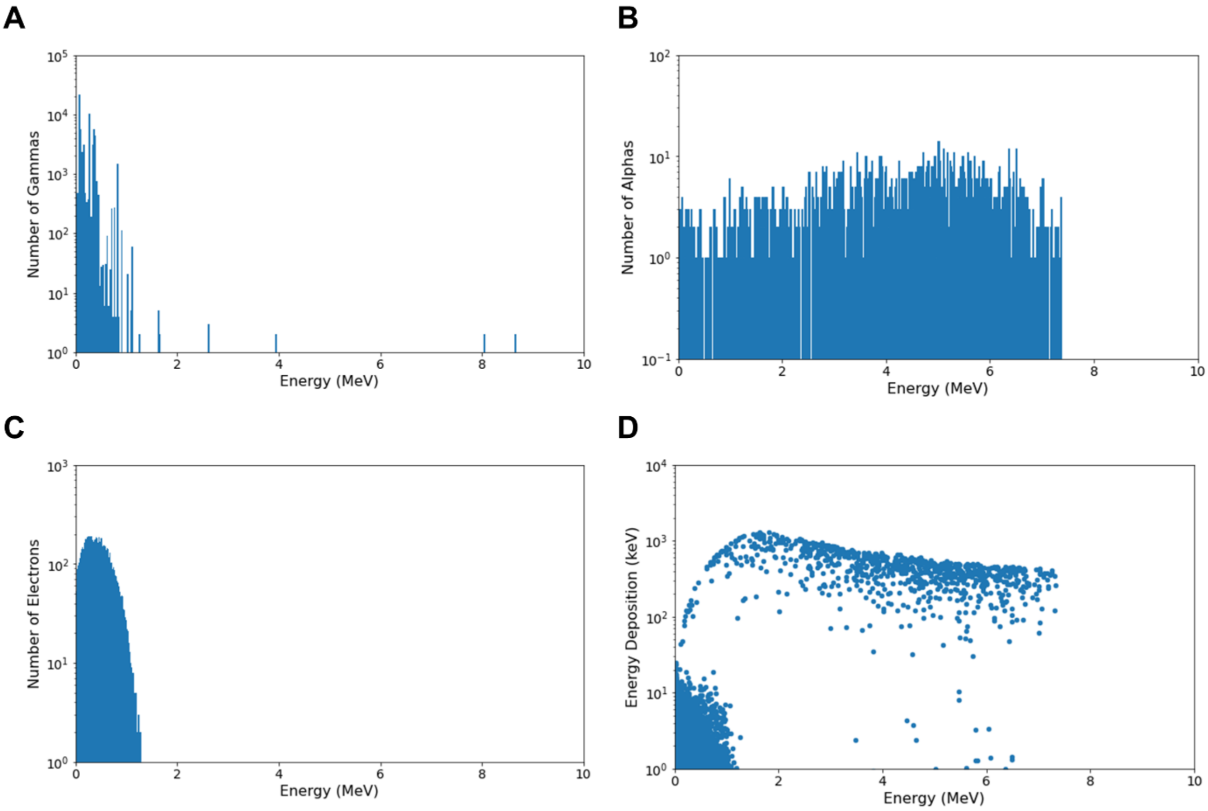


Supplemental figure 6. $^{223}$Ra phase space: A) Gamma spectrum. B) Alpha spectrum. C) Electron spectrum. C) Energy deposition vs. energy for all particles in the phase space.

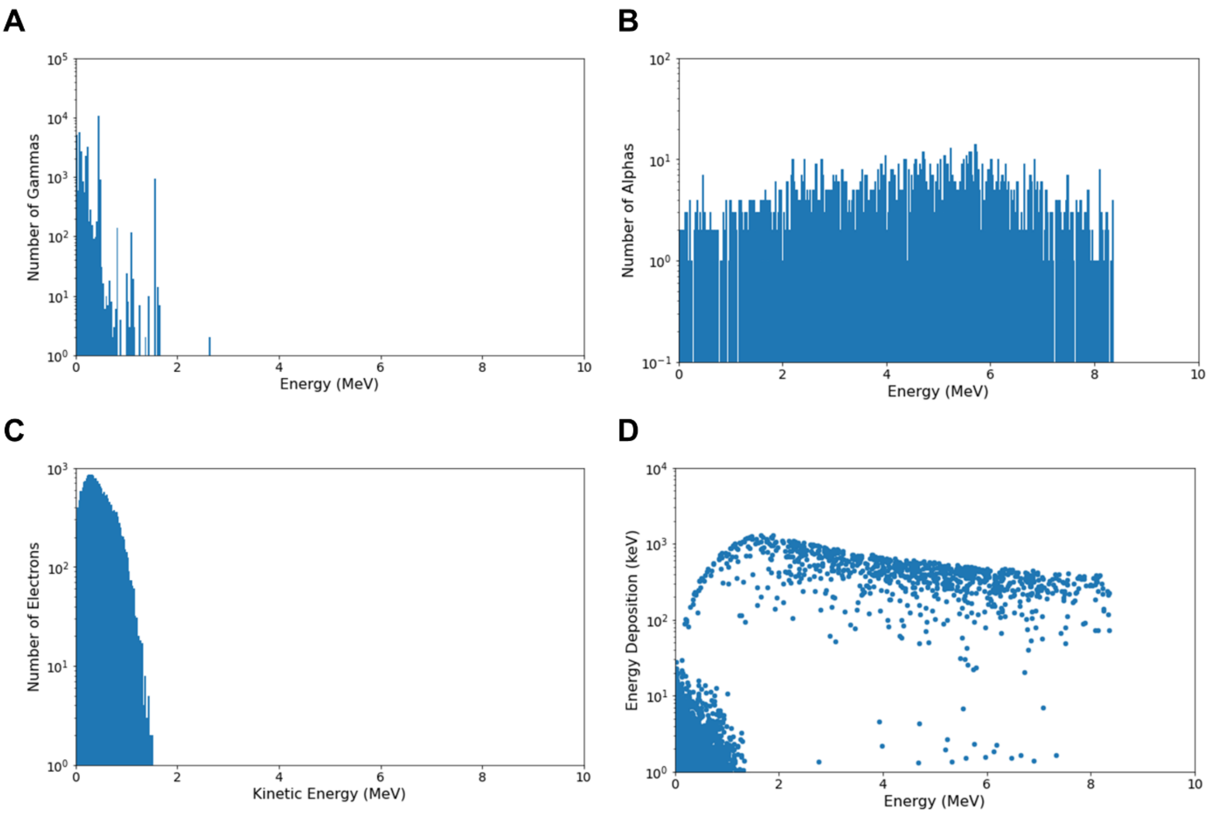


Supplemental figure 7. $^{225}$Ac phase space: A) Gamma spectrum. B) Alpha spectrum. C) Electron spectrum. C) Energy deposition vs. energy for all particles in the phase space.
